# Carpal Kinematics in the Normal, Scapholunate Ligament Deficient, and Surgically Reconstructed Wrist

**DOI:** 10.1002/jor.26049

**Published:** 2025-02-02

**Authors:** Xin Zhang, Stephen K. Tham, Bruno Crepaldi, Eugene T. Ek, David McCombe, David Charles Ackland

**Affiliations:** ^1^ Department of Biomedical Engineering University of Melbourne Parkville Victoria Australia; ^2^ Department of Plastic and Hand Surgery St Vincent's Hospital Fitzroy Victoria Australia; ^3^ Department of Orthopaedic Surgery, Division of Hand Surgery Dandenong Hospital Dandenong Victoria Australia; ^4^ Hand and Wrist Biomechanics Laboratory O'Brien Institute Fitzroy Victoria Australia

**Keywords:** carpal, kinematic model, scapholunate instability, scapholunate interosseous ligament, stereophotogrammetric analysis

## Abstract

The objective of this study was to evaluate scaphoid, lunate and capitate kinematics after disruption to the primary and secondary scapholunate ligamentous stabilizers, and to assess the effectiveness of scapholunate ligament reconstruction in restoring carpal kinematics post‐operatively. Seven upper extremities were harvested, and the scapholunate interosseous ligament (SLIL) was divided. Specimens were mounted onto a computer‐controlled dynamic wrist simulator, and simulations of flexion‐extension, radial‐ulnar deviation, and dart‐thrower's motion were undertaken by simulated force application to the wrist tendons. Three‐dimensional kinematics of the scaphoid, lunate and capitate were measured using bi‐plane X‐ray fluoroscopy in the native and ligament deficient state. The SLIL was then reconstructed by either dorsal transarticular loop tenodesis (DTLT), or by the three‐ligament tenodesis (3LT) technique, and re‐evaluated. SLIL deficiency resulted in significant differences in carpal kinematics compared to that in the healthy wrist across all wrist motions (*p* < 0.05). The DTLT procedure corrected increased scaphoid ulnar deviation and pronation in the SLIL deficient wrist, but did not significantly improve scaphoid flexion or volar translation of the scaphoid. The 3LT reconstructive technique restored scaphoid flexion and ulnar deviation but did not correct pronation, the increased lunate extension, nor the volar and ulnar translation observed in the ligament deficient wrist. Three‐dimensional scaphoid, lunate and capitate motion depends on SLIL integrity, with tears to this ligament resulting in pathological kinematics, which may be partially mitigated with DTLT and 3LT surgical reconstruction. These findings suggest that this surgical reconstruction of the SLIL may not mitigate long‐term degenerative joint conditions at the wrist.

## 1 Introduction

Stability of the scapholunate joint is primarily achieved via the scapholunate interosseous ligament (SLIL), with several other ligamentous structures providing secondary stability [1]. Injuries to the SLIL are common [2, 3], and if left untreated, can lead to degenerative joint disease in a pattern known as scapholunate advanced collapse [4]. Intercarpal arthrodesis for the treatment of SLIL injuries results in loss of wrist motion [5, 6], alters normal joint kinematics and contact mechanics [7, 8] and has a significant rate of nonunion [9], often requiring further surgery. In wrists without arthritic changes but with a reducible carpal malalignment, reconstructive surgery can be performed with the aim of restoring normal scapholunate alignment, relieving pain, and improving wrist function. Soft‐tissue reconstructive techniques have gained popularity as they are thought to better preserve intercarpal joint kinematics, though there is a paucity of carpal bone motion data that demonstrates this [10].

Established reconstructive techniques include soft‐tissue procedures such as capsulodesis [11, 12, 13], ligament reconstruction using tendon graft or bone‐ligament‐bone autografts [14, 15, 16], establishing a pseudoarthrosis between the scaphoid and lunate using headless compression screws [17], and limited intercarpal fusion [18]. Tenodesis procedures, in which a tendon graft is weaved across the scapholunate joint to reconstruct the deficient SLIL, are thought to result in better multiplanar control of the scaphoid compared to capsulodesis procedures [13, 19]. A modification of the Brunelli procedure [20], the three‐ligament tenodesis (3LT), has been used to treat chronic reducible scapholunate instability [21]. The technique aims to reconstruct the dorsal SLIL (d‐SLIL) while augmenting the palmar scaphotrapeziotrapezoid (STT) and dorsal radiocarpal (DRC) ligaments without the need to tether the tendon weave to the distal radius. However, the reported success rates of soft‐tissue reconstruction vary [22].

An alternative technique for reconstructing the d‐SLIL was developed by the senior clinician. The dorsal transarticular loop tenodesis (DTLT) technique directly reconstructs the d‐SLIL by the passage of two tendon grafts measuring 2 mm wide through two burr holes on the adjacent dorsal surfaces of the scaphoid and lunate. The aims of this study were twofold. First, to employ a validated dynamic cadaveric wrist testing apparatus, together with bi‐plane X‐ray fluoroscopy, to evaluate scaphoid, lunate and capitate kinematics after disruption to the primary and secondary scapholunate ligamentous stabilizers; and second, to evaluate the effectiveness of both the 3LT technique and DTLT methods in restoring carpal kinematics post‐operatively. It was hypothesized that the DTLT method would reproduce normal carpal kinematics more effectively than the 3LT method as the DTLT directly reconstructs the d‐SLIL, whereas the 3LT indirectly reconstructs the d‐SLIL by the passage of one length of tendon graft from the scaphoid looping around the dorsal extrinsic ligament.

## 2 Materials and Methods

### 2.1 Specimen Preparation

Seven upper extremities were harvested from five fresh‐frozen human cadavers (mean age: 82.2 ± 12.5 years; Type I lunate: 6 specimens; Type II lunate: 1 specimen). This sample size of convenience was chosen based on a similar‐designed wrist kinematics study that identified significant differences in carpal bone motion relative to global wrist motion [23]. The wrist of each specimen was radiographically screened for previous trauma or joint degeneration conditions. Each specimen was disarticulated at the elbow while preserving the integrity of the proximal radioulnar joint. The wrist prime movers, which included the flexor carpi ulnaris (FCU) and radialis (FCR), extensor carpi radialis brevis (ECR‐B) and longus, extensor carpi ulnaris, and abductor pollicis longus, were detached from their proximal origins and dissected up to the musculotendinous junction.

A portion of the tendon measuring 3 mm wide and 10 cm long was split from the FCR and ECR‐B tendons and set aside for reconstructive surgery. All other tendons were trimmed, grasped with custom wire‐woven sleeves and suture (Ethibond Excel, Size 2‐0; Ethicon, New Jersey, USA), and wrapped in saline‐soaked gauze. All intrinsic forearm muscles, skin, and other subcutaneous tissues were then excised. Each specimen was then held in neutral pronation‐supination by drilling two Steinmann pins transversely through the shafts of the radius and ulna at the mid‐forearm level.

The wrist capsule was exposed through a ligament‐sparing dorsal capsulotomy [24]. To facilitate kinematic measurement using a custom bi‐plane fluoroscopy system, a cluster of three radio‐opaque markers varying in size from 1.0 to 1.8 mm were embedded into each of the dorsal cortices of the scaphoid, lunate, capitate, and distal radius. Retro‐reflective marker triads were attached to the radius and third metacarpal to facilitate real‐time tracking of global wrist motion using a 4‐camera high‐speed video motion analysis system (Vero, Vicon, Oxford Metrics, UK). Specimens were then scanned using computed tomography (CT) (Siemens Biograph mCT, Berlin, Germany) with a 0.1 mm slice thickness. Volumetric bone models were then generated from the CT scans and the radio‐opaque marker locations registered to anatomical reference frames (Mimics, Materialise, Belgium).

### 2.2 Surgical Reconstruction of the SLIL

**Figure 1:**
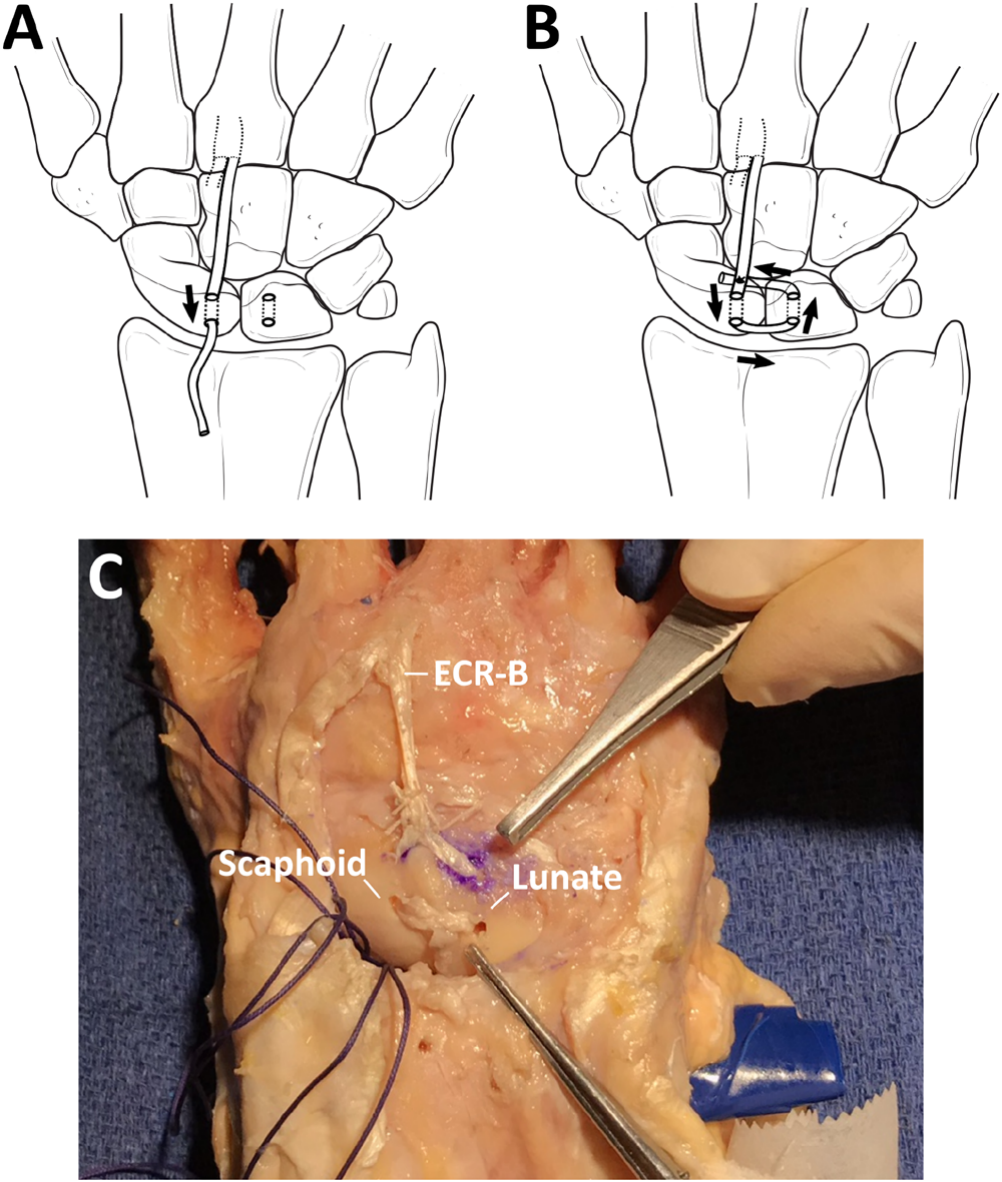
Diagram illustrating the DTLT procedure for reconstruction of the scapholunate ligament deficient wrist, performed on the neutrally positioned wrist. Reconstruction was achieved by realigning and reducing the scapholunate joint, then stabilizing it with two Kirschner wires (K‐wire) placed across the scapholunate interval and one across the scaphocapitate (SC) joint. Two parallel connected burr holes, each measuring 2 mm in width (A), were created on the dorsum and adjacent surfaces of both the scaphoid and lunate in the region of the d‐SLIL insertion. The previously prepared ECR‐B graft was then passed dorsally, initially through the scaphoid and then the lunate in a box‐shaped fashion. The tendon graft was finally sutured back on itself, completing a transarticular passage and resulting in two parallel tendon grafts across the dorsal scapholunate interval, after which the K‐wires were removed (B). A photograph of the technique performed on a representative cadaveric specimen is also given (C).

**Figure 2:**
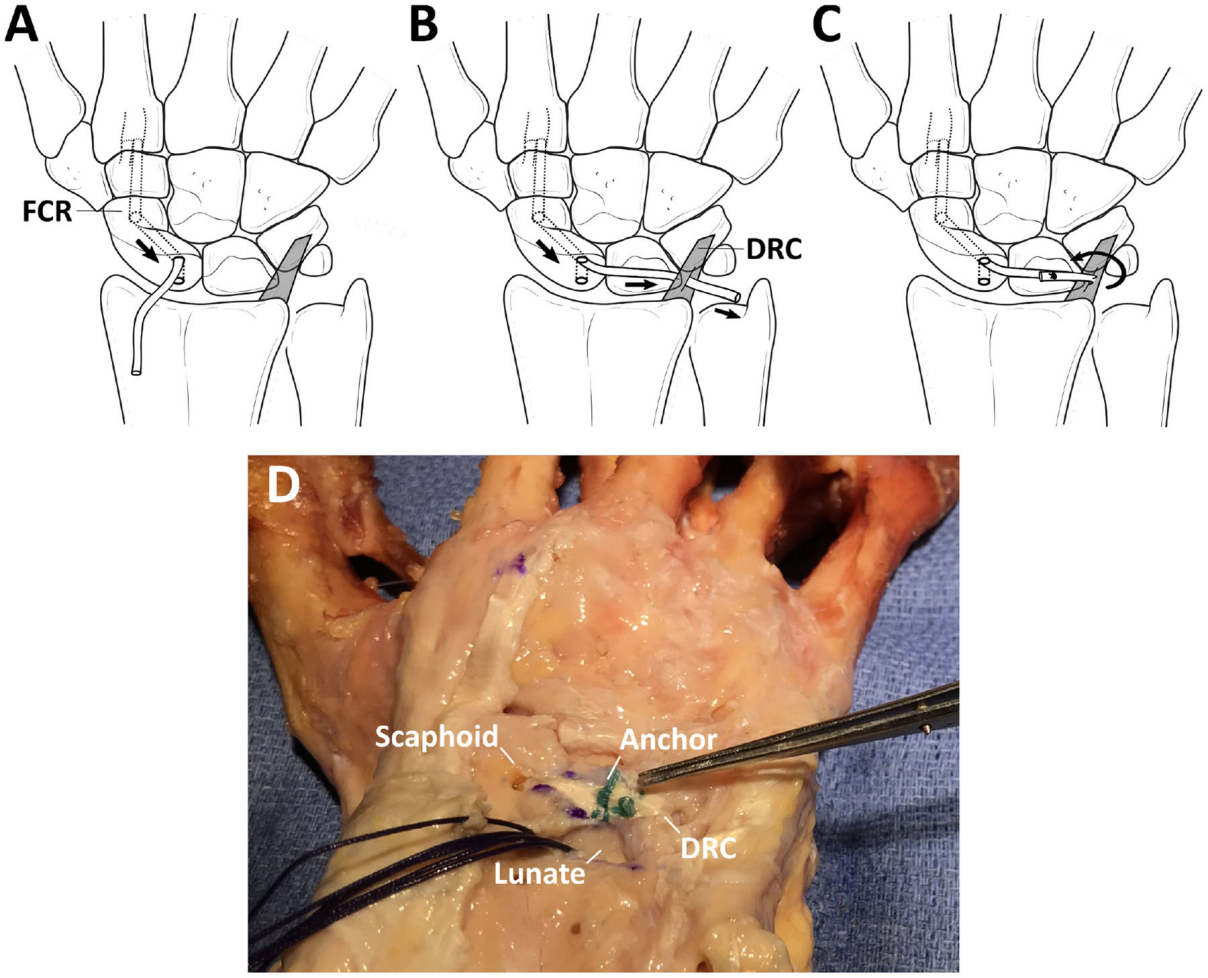
Diagram illustrating the 3LT procedure for reconstruction of the scapholunate deficient wrist. A 3 mm wide, obliquely directed tunnel through the scaphoid was made under fluoroscopic guidance (A). This hole entered the scaphoid proximally and dorsally from one of the drill holes created for the DTLT technique and exited volarly at the scaphoid tubercle. The previously harvested FCR tendon strip was then passed through the tunnel. Sutures were used to assist the passage of the tendon strip. The tendon graft was then guided through a slit made on the distal and ulnar portion of the remaining DRC ligament, allowing it to be folded back onto the lunate (B). By using the DRC as a pivot, the tendon graft was then tightened until satisfactory reduction of the scapholunate joint was achieved, after which two scapholunate and one scaphocapitate wires K‐wires were inserted to maintain the reduction. The FCR graft was secured to the dorsal lunate using a radiolucent suture anchor (JuggerKnot Mini 1.0 mm; Zimmer Biomet, Indiana, USA), and the K‐wires were then removed (C). K‐Y jelly was applied to promote gliding between the ligament graft and the dorsal rim of the distal radius, mimicking the moist, low‐friction ligament gliding that occurs in vivo. A photograph of the technique performed on a representative cadaveric specimen is also given (D).

The SLIL, including its volar (v‐), membranous (m‐), and dorsal (d‐) subregions, and the secondary SL stabilizers, including the radioscaphocapitate (RSC), long radiolunate (LRL), palmar STT, dorsal intercarpal (DIC) ligaments, and approximately 40% of the DRC ligament, were sequentially divided under loupe magnification. The DRC was only partially retained due to its use in one of the subsequent tenodesis reconstructions. The DTLT procedure was first performed to reconstruct the scapholunate ligament deficient wrist (Figure 1), and the wrist biomechanically tested. The 3LT procedure [21] was then carried out after removing the ECR‐B graft and supporting sutures from the DTLT reconstruction (Figure 2), and the wrist was subsequently tested again. The specimen was completely dissected at the conclusion of each experiment to confirm all intended ligament resections and that subsequent surgical reconstruction had been performed correctly.

### 2.3 Biomechanical Testing

**Figure 3:**
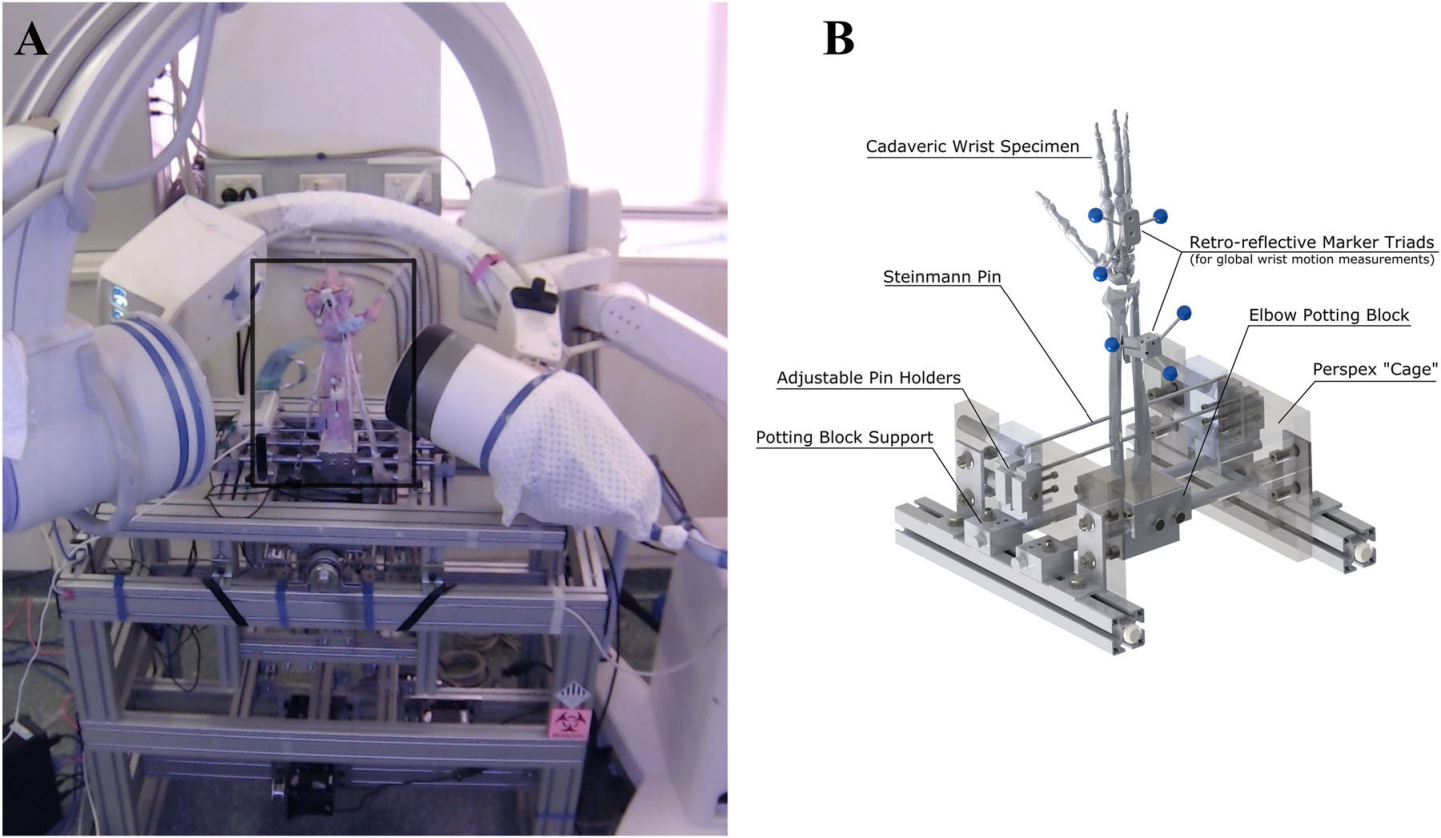
Dynamic cadaveric wrist testing apparatus (A), and specimen mounting platform (B), used to simulate dynamic motion of the wrist. Cadaveric wrist specimens had retro‐reflective marker triads inserted into the radius and third metacarpal so that global wrist motion could be measured using a video motion analysis system during testing. Wrist muscle‐tendon forces were then controlled in real‐time to reproduce the desired dynamic wrist motion trajectories. Specimens were rigidly fixed to an elbow potting block, which was mounted on a support and surrounded by a rigid Perspex cage for additional support to the mounting fixtures. Steinman pins passing through the radius and ulna to adjustable pin holders were employed to provide additional support to each specimen. Reprinted with permission from Elsevier.

A previously validated computer‐controlled dynamic wrist simulator was employed to replicate wrist motion by simulated muscle force application (Figure 3) [25]. The proximal radius and ulna of each specimen were first cemented to a potting fixture and mounted. Each of the six wrist muscles was then connected to an electromechanical actuator, with a uniaxial load cell attached in‐line to provide tendon force measurement during testing (IMM‐K20, Dacell, Korea). To achieve the desired wrist motion, a closed‐loop control system was employed using global wrist position as input, which was measured using the four‐camera video motion analysis system. This allowed muscle forces, which included co‐contraction, to be calculated and updated by the controller in real‐time to achieve the desired wrist motion trajectories [25]. The dynamic wrist simulator can reproduce global wrist motion within 0.5° of a target trajectory profile in both healthy and scapholunate ligament deficient wrists and has demonstrated repeatability and reproducibility both within 0.1° between successive motion cycles in the same specimen and across different specimens.

Wrist motion simulations were then performed, which included flexion‐extension motion (FEM) to a maximum of 30° in each direction, radial‐ulnar deviation (RUD) to a maximum of 20° in each direction, and the superposition of the two aforementioned motion trajectories (i.e., ulnar flexion and radial extension), as an approximation of the dart‐thrower's motion (DTM) oriented 33.7° to the sagittal plane. To achieve this, a sinusoidal motion profile of a 20‐s period was employed, and three consecutive cycles of motion were performed in each wrist simulation. The global wrist motion was captured by the video motion analysis system at a sampling rate of 100 Hz, and these data were fed into the dynamic wrist simulator to control wrist position in real‐time. Wrist joint positions, including the definition of the neutral position, were defined from the position of the 3rd metacarpal relative to the radius as per ISB recommendations [26]. For each specimen, biomechanical testing was first carried out in its healthy, intact state, then repeated after division of the primary and secondary scapholunate supporting ligaments, after reconstruction using the DTLT procedure, and then finally, after 3LT with the prior reconstruction removed. The surgical goals of these procedures were to reduce the scaphoid‐lunate gap and restore the scapholunate angle.

During wrist motion simulations, scaphoid, lunate and capitate rotations and translations were simultaneously measured using bi‐plane X‐ray fluoroscopy and marker‐based Roentgen Stereophotogrammetric Analysis. To achieve this, a custom bi‐plane X‐ray fluoroscopy system comprising two C‐arms X‐ray units (OEC FlexiView 8800; GE Healthcare, USA and Fluoroscan Insight II; Hologic, USA) was retro‐fitted with synchronized machine vision cameras (acA1920‐150um and acA1300‐200um; Basler, Germany). After distortion correction and calibration, the two C‐arms were operated under continuous fluoroscopy mode, and bi‐planar image data of the simulated wrist motions were acquired at 25Hz. The system measured rotations and translations to an accuracy of 0.18° and 0.05 mm, respectively [25]. All carpal rotations were measured with respect to the radius and reported in Euler angles using a Z‐X′‐Y″ sequence (flexion/extension‐radial/ulnar deviation‐pronation/supination). Translations were recorded from the motion of each carpal bone's centroid.

### 2.4 Data Analysis

Scaphoid, lunate, and capitate kinematics during FEM, RUD, and DTM following each surgical reconstruction technique were compared with those in the healthy, intact state and after ligament division. The sinusoidal motion profile for each wrist simulation was segmented into four equal quarters to represent distinct phases of movement: from the neutral (0%) position to the positive extremum (25%), returning to neutral (50%), moving to the negative extremum (75%), and finally returning to neutral (100%). Specifically, the first and third quarters of the motion profile for FEM were defined for forward movements corresponding to wrist flexion and extension, respectively. Similarly, for RUD, the first and third quarters were analyzed for ulnar and radial deviations, respectively; and for DTM, the first and third quarters corresponded to ulnar flexion and radial extension. Mean carpal bone kinematics across these specific regions of the motion trajectory were statistically compared across different specimen conditions (i.e. normal, ligament deficient, surgically reconstructed using 3LT, and surgically reconstructed with DTLT) using $L^{2}$‐norm‐based one‐way functional data analysis of variance [27], with the significance level defined as *p* < 0.05.

## 3 Results

### 3.1 Ligament Deficient Wrist

**Figure 4:**
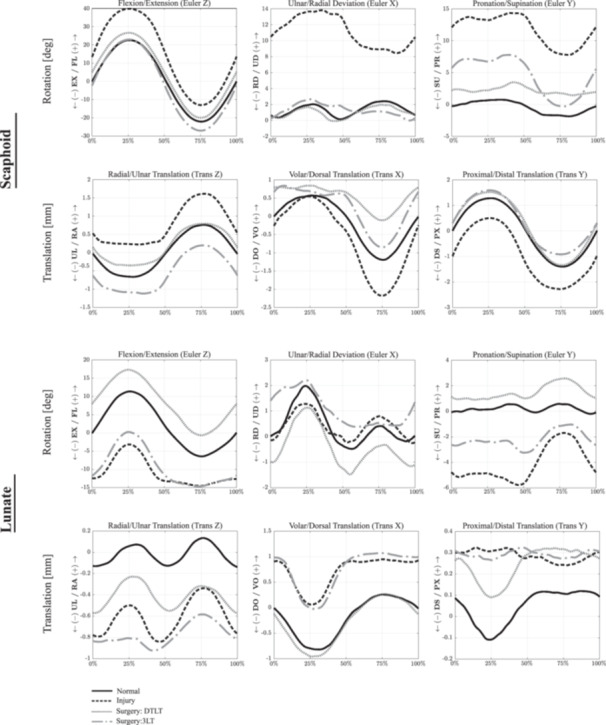
Mean scaphoid and lunate kinematics during wrist flexion and extension in the normal, ligament deficient and surgically reconstructed wrist following the DTLT and 3LT procedures. Given are the three Euler angle rotations (degrees), including flexion–extension (*Z*), ulnar‐radial deviation (*X*), and pronation‐supination (*Y*), as well as bone translations (mm). Data are given as a percentage of the sinusoidal wrist flexion–extension motion cycle. Symbol definitions are as follows: EX, extension; FL, flexion; PR, pronation; RD, radial deviation; SU, supination; UD, ulnar deviation.

**Figure 5:**
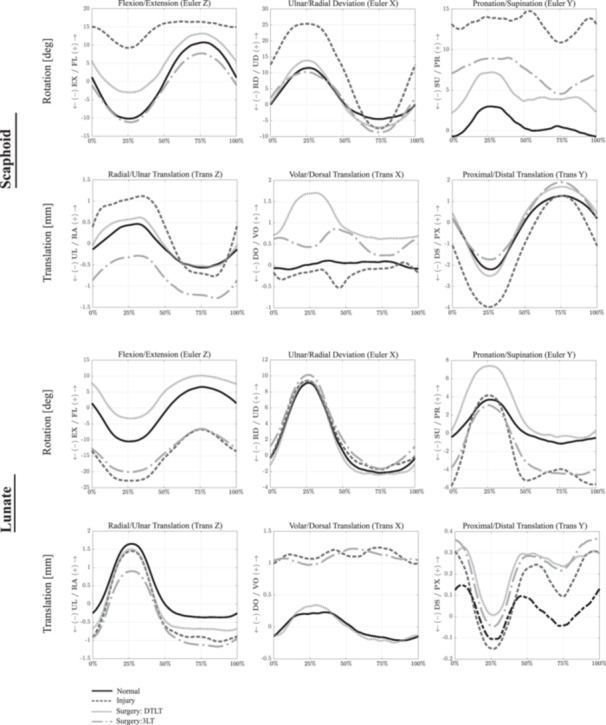
Mean scaphoid and lunate kinematics during wrist radial‐ulnar deviation in the normal, ligament deficient and surgically reconstructed wrist following the DTLT and 3LT procedures. Given are the three Euler angle rotations (degrees), including flexion–extension (*Z*), ulnar‐radial deviation (*X*) and pronation‐supination (*Y*), as well as bone translations (mm). Data are given as a percentage of the sinusoidal wrist ulnar‐radial deviation motion cycle. See Figure 4 Caption for more information.

Dividing the SLIL, RSC, LRL, palmar STT, DIC, and parts of DRC resulted in significant differences in scaphoid, lunate and capitate kinematics compared to that in the healthy wrist across all wrist motions (*p* < 0.05). The scaphoid had greater flexion, ulnar deviation, pronation, and increased translation in the radial and distal directions during wrist flexion (*p* < 0.05) (Figure 4; Table 1). After ligament sectioning, the largest, significant increases in scaphoid flexion, ulnar deviation, and distal translation were observed during wrist ulnar deviation (mean difference: 18.5°, 14.3°, and 1.7 mm, respectively) (*p* < 0.01) (Figure 5), while the largest increases in scaphoid pronation were observed during wrist flexion (mean difference: 13.0°) (*p* < 0.01).

**Table 1:** Mean differences and *p*‐values for scaphoid Euler angle rotations (in degrees) and translations (in mm) across normal, ligament deficient, and surgically reconstructed wrists.

		Rotation [deg]									Translation [mm]								
		Flexion			Ulnar deviation			Pronation			Radial			Volar			Proximal		
		Mean diff.	95% CI	*p*‐value	Mean diff.	95% CI	*p*‐value	Mean diff.	95% CI	*p*‐value	Mean diff.	95% CI	*p*‐value	Mean diff.	95% CI	*p*‐value	Mean diff.	95% CI	*p*‐value
Flexion	Injury versus Normal	17.0	[13.0, 21.1]	< 0.001***	11.3	[8.2, 14.3]	< 0.001***	13.0	[10.2, 15.8]	< 0.001***	0.8	[0.3, 1.2]	0.006**	−0.1	[−1.3, 1.1]	0.857	−0.8	[−1.3, −0.4]	< 0.001***
	DTLT versus Normal	4.7	[0.4, 8.9]	0.050*	−0.1	[−5.2, 5.0]	0.957	1.9	[−1.8, 5.6]	0.345	0.2	[−0.2, 0.7]	0.377	0.4	[0.1, 0.7]	0.008**	0.3	[−0.2, 0.7]	0.275
	3LT versus Normal	0.7	[−4.3, 5.6]	0.679	0.4	[−5.1, 5.9]	0.893	6.7	[3.5, 9.9]	< 0.001***	−0.5	[−1.1, 0.1]	0.156	0.4	[0.2, 0.6]	< 0.001***	0.3	[−0.1, 0.7]	0.275
Extension	Injury versus Normal	10.9	[3.7, 18.0]	0.005**	8.5	[5.9, 11.1]	< 0.001***	11.6	[9.3, 13.9]	< 0.001***	0.7	[−0.3, 1.7]	0.204	−0.7	[−3.9, 2.5]	0.671	−1.1	[−1.8, −0.4]	0.006**
	DTLT versus Normal	2.8	[0.5, 5.2]	0.025*	−0.2	[−4.3, 3.9]	0.905	3.3	[0.2, 6.4]	0.057	0.1	[−0.2, 0.4]	0.668	1.0	[0.7, 1.2]	< 0.001***	0.1	[−0.4, 0.6]	0.626
	3LT versus Normal	−5.2	[−9.9, −0.5]	0.068	−0.4	[−4.6, 3.7]	0.716	3.4	[1.6, 5.2]	< 0.001***	−0.5	[−0.9, −0.1]	0.018*	0.4	[0.2, 0.6]	< 0.001***	0.3	[−0.2, 0.7]	0.312
Ulnar deviation	Injury versus Normal	18.6	[11.9, 25.2]	< 0.001***	14.3	[10.5, 18.1]	< 0.001***	12.2	[9.2, 15.3]	< 0.001***	0.7	[0.0, 1.3]	0.060	−0.2	[−1.6, 1.3]	0.826	−1.7	[−2.2, −1.2]	< 0.001***
	DTLT versus Normal	6.3	[1.7, 10.8]	0.012*	1.8	[−4.8, 8.3]	0.599	3.9	[0.3, 7.6]	0.052	0.2	[−0.2, 0.6]	0.356	1.3	[0.9, 1.8]	< 0.001***	−0.1	[−0.8, 0.5]	0.570
	3LT versus Normal	−0.5	[−5.3, 4.3]	0.787	0.3	[−5.8, 6.3]	0.737	6.8	[4.5, 9.2]	< 0.001***	−0.6	[−1.0, −0.2]	0.017*	0.6	[0.3, 0.9]	0.002**	0.1	[−0.4, 0.7]	0.477
Radial deviation	Injury versus Normal	9.4	[3.5, 15.2]	0.002**	2.4	[0.1, 4.6]	< 0.001***	12.8	[10.8, 14.9]	< 0.001***	0.1	[−0.3, 0.4]	0.326	−0.2	[−1.9, 1.4]	0.814	−0.4	[−0.7, −0.1]	0.003**
	DTLT versus Normal	3.3	[0.6, 5.9]	0.030*	−2.0	[−4.6, 0.6]	0.199	3.7	[1.7, 5.7]	0.002**	0.0	[−0.4, 0.4]	0.928	0.6	[0.4, 0.8]	< 0.001***	0.4	[0.1, 0.7]	0.036*
	3LT versus Normal	−2.4	[−6.1, 1.2]	0.270	−2.1	[−5.3, 1.0]	0.185	6.0	[4.0, 8.1]	< 0.001***	−0.6	[−1.0, −0.2]	0.010*	0.4	[0.2, 0.7]	0.004**	0.4	[0.0, 0.7]	0.065
DTM (Ulnar Flexion)	Injury versus Normal	15.8	[12.2, 19.5]	< 0.001***	11.9	[7.7, 16.1]	< 0.001***	12.1	[9.8, 14.4]	< 0.001***	0.6	[0.1, 1.1]	0.029*	0.3	[−0.6, 1.3]	0.554	−1.2	[−1.7, −0.8]	< 0.001***
	DTLT versus Normal	6.6	[0.8, 12.4]	0.041*	1.4	[−5.2, 8.0]	0.651	2.6	[−2.1, 7.4]	0.324	0.3	[−0.2, 0.8]	0.318	1.3	[0.9, 1.7]	< 0.001***	0.0	[−0.7, 0.7]	0.642
	3LT versus Normal	−0.2	[−4.6, 4.3]	0.829	0.1	[−5.5, 5.7]	0.744	7.6	[4.5, 10.6]	< 0.001***	−0.6	[−1.1, 0.0]	0.076	0.5	[0.3, 0.6]	< 0.001***	0.2	[−0.4, 0.8]	0.528
DTM (Radial Extension)	Injury versus Normal	5.0	[−0.7, 10.8]	0.045*	2.5	[0.5, 4.5]	< 0.001***	8.7	[7.2, 10.2]	< 0.001***	−0.2	[−0.4, 0.0]	0.040*	−0.9	[−4.0, 2.2]	0.576	−0.3	[−0.6, −0.1]	< 0.001***
	DTLT versus Normal	2.7	[0.2, 5.1]	0.064	−1.5	[−3.8, 0.7]	0.238	2.4	[0.7, 4.1]	0.031*	0.0	[−0.3, 0.3]	0.808	0.8	[0.5, 1.0]	< 0.001***	0.4	[0.0, 0.7]	0.081
	3LT versus Normal	−3.8	[−7.2, −0.3]	0.064	−0.8	[−3.4, 1.7]	0.551	3.5	[1.9, 5.1]	< 0.001***	−0.6	[−1.0, −0.1]	0.030*	0.5	[0.1, 0.8]	0.015*	0.3	[−0.1, 0.6]	0.193

*Note:* Displayed are the mean differences for the entirety of specified dynamic wrist motions between ligament deficient (injury) wrists compared to normal and each surgical reconstruction technique (DTLT and 3LT) compared to normal. “Flexion,” “ulnar deviation,” and “ulnar flexion” correspond to the first quarter (0%–25%) of the wrist motion cycle, while “extension,” “radial deviation,” and “radial extension” correspond to the third quarter (50%–75%) of the cycle. Given are the mean differences between the ligament deficient (injury) wrist versus normal, DTLT reconstruction versus normal, and 3LT reconstruction versus normal. Data are provided for flexion/extension, ulnar/radial deviation, and dart‐thrower's motion of the wrist. Positive *Z*, *X*, and *Y* rotations represent flexion, ulnar deviation, and pronation, respectively. Negative *Z*, *X*, *Y* rotations represent extension, radial deviation, and supination, respectively. Positive *Z*, *X*, *Y* translations are in the radial, volar, and proximal directions, respectively. Negative *Z*, *X*, *Y* translations are in the ulnar, dorsal, and distal directions, respectively. All angles and translations are reported with respect to the radius. Mean differences along with a 95% confidence interval (CI) are given for each comparison. The asterisk indicates *p* < 0.05.

After ligament sectioning, the lunate was significantly more extended (mean difference: 15.0°, *p* < 0.001) and supinated (mean difference: 5.0°, *p* < 0.001) during wrist flexion, and this was accompanied by significantly greater lunate translations (*p* < 0.001) (Table 2). The capitate was significantly more flexed (mean difference: 1.6°, *p* < 0.001) and pronated (mean difference: 4.7°, *p* < 0.001) during wrist radial deviation, while significant dorsal (mean difference: 2.2 mm, *p* < 0.001) and proximal translation (mean difference: 1.5mm, *p* < 0.001) was observed during wrist flexion (Table 3). For further details of kinematics in the ligament deficient wrist, see the previously published study by Zhang et al. [25].

**Table 2:** Mean differences and *p*‐values for lunate Euler angle rotations (in degrees) and translations (in mm) across normal, ligament deficient, and surgically reconstructed wrists.

		Rotation [deg]									Translation [mm]								
		Flexion			Ulnar deviation			Pronation			Radial			Volar			Proximal		
		Mean diff.	95% CI	*p*‐value	Mean diff.	95% CI	*p*‐value	Mean diff.	95% CI	*p*‐value	Mean diff.	95% CI	*p*‐value	Mean diff.	95% CI	*p*‐value	Mean diff.	95% CI	*p*‐value
Flexion	Injury versus Normal	−15.0	[−19.7, −10.3]	< 0.001***	−0.3	[−1.8, 1.2]	0.687	−5.0	[−5.8, −4.3]	< 0.001***	−0.6	[−0.9, −0.4]	< 0.001***	1.0	[0.5, 1.4]	< 0.001***	0.3	[0.2, 0.5]	< 0.001***
	DTLT versus Normal	6.6	[0.1, 13.2]	0.091	−0.6	[−1.4, 0.3]	0.276	0.9	[−0.2, 2.0]	0.230	−0.4	[−0.6, −0.1]	0.036*	−0.1	[−0.6, 0.4]	0.720	0.2	[0.1, 0.4]	0.025*
	3LT versus Normal	−12.4	[−16.3, −8.5]	< 0.001***	1.2	[−0.3, 2.8]	0.165	−2.5	[−3.9, −1.1]	0.003**	−0.8	[−1.2, −0.3]	0.003**	1.0	[0.6, 1.5]	< 0.001***	0.3	[0.2, 0.5]	< 0.001***
Extension	Injury versus Normal	−10.7	[−16.1, −5.4]	< 0.001***	0.4	[−1.5, 2.2]	0.729	−3.6	[−6.3, −0.9]	0.013*	−0.5	[−0.9, −0.2]	0.005**	0.8	[0.4, 1.3]	< 0.001***	0.2	[0.0, 0.3]	0.040*
	DTLT versus Normal	6.0	[0.6, 11.4]	0.064	−0.5	[−1.3, 0.3]	0.314	1.5	[−0.2, 3.2]	0.143	−0.5	[−0.6, −0.3]	< 0.001***	0.0	[−0.5, 0.5]	0.819	0.2	[0.1, 0.4]	0.014*
	3LT versus Normal	−11.0	[−13.3, −8.7]	< 0.001***	0.9	[−0.5, 2.3]	0.272	−2.5	[−4.1, −0.9]	0.008**	−0.7	[−1.1, −0.3]	0.003**	1.0	[0.7, 1.4]	< 0.001***	0.2	[0.0, 0.4]	0.028*
Ulnar deviation	Injury versus Normal	−13.7	[−19.9, −7.4]	< 0.001***	0.5	[−0.4, 1.5]	0.358	−1.7	[−3.1, −0.2]	0.002**	−0.4	[−0.7, 0.0]	0.051	1.0	[0.6, 1.4]	< 0.001***	0.0	[−0.1, 0.2]	0.392
	DTLT versus Normal	7.6	[−1.2, 16.3]	0.159	−0.3	[−1.3, 0.6]	0.648	2.9	[1.9, 3.9]	< 0.001***	−0.4	[−0.7, −0.1]	0.055	0.0	[−0.5, 0.6]	0.830	0.2	[0.0, 0.3]	0.104
	3LT versus Normal	−10.8	[−13.6, −8.0]	< 0.001***	1.0	[0.3, 1.7]	0.035*	−1.6	[−3.6, 0.3]	0.115	−0.8	[−1.1, −0.5]	< 0.001***	1.0	[0.7, 1.3]	< 0.001***	0.1	[0.0, 0.3]	0.025*
Radial deviation	Injury versus Normal	−14.1	[−19.2, −9.0]	< 0.001***	0.6	[−1.2, 2.5]	0.562	−4.0	[−4.9, −3.1]	< 0.001***	−0.6	[−0.8, −0.3]	< 0.001***	1.3	[0.8, 1.7]	< 0.001***	0.2	[0.1, 0.3]	0.004**
	DTLT versus Normal	4.5	[−1.1, 10.2]	0.174	−0.1	[−1.2, 0.9]	0.658	0.8	[−0.3, 1.8]	0.235	−0.4	[−0.6, −0.1]	0.016*	0.0	[−0.6, 0.6]	0.962	0.3	[0.2, 0.4]	< 0.001***
	3LT versus Normal	−13.7	[−16.9, −10.6]	< 0.001***	1.4	[0.3, 2.5]	0.033*	−3.3	[−4.3, −2.3]	< 0.001***	−0.7	[−1.1, −0.4]	0.001**	1.3	[1.0, 1.7]	< 0.001***	0.2	[0.1, 0.4]	0.006**
DTM (ulnar flexion)	Injury versus Normal	−13.5	[−19.4, −7.7]	< 0.001***	1.5	[0.3, 2.8]	0.014*	−2.1	[−3.0, −1.1]	< 0.001***	−0.1	[−0.5, 0.3]	0.249	0.9	[0.6, 1.2]	< 0.001***	0.1	[0.0, 0.2]	0.158
	DTLT versus Normal	10.0	[0.2, 19.7]	0.088	−0.4	[−1.0, 0.3]	0.358	1.1	[0.1, 2.2]	0.078	−0.4	[−0.7, 0.0]	0.102	−0.1	[−0.6, 0.5]	0.801	0.1	[−0.1, 0.3]	0.333
	3LT versus Normal	−11.0	[−15.8, −6.2]	< 0.001***	2.0	[1.6, 2.4]	< 0.001***	−1.7	[−3.6, 0.2]	0.142	−0.7	[−1.0, −0.3]	< 0.001***	0.9	[0.5, 1.3]	< 0.001***	0.1	[0.0, 0.3]	0.058
DTM (radial extension)	Injury versus Normal	−11.7	[−17.7, −5.7]	< 0.001***	1.5	[−0.6, 3.5]	0.194	−2.2	[−4.0, −0.3]	0.004**	−0.4	[−0.7, 0.0]	0.047*	0.9	[0.4, 1.4]	< 0.001***	0.1	[0.0, 0.2]	0.017*
	DTLT versus Normal	5.0	[−0.5, 10.5]	0.132	−0.4	[−1.5, 0.6]	0.340	1.5	[0.1, 2.8]	0.055	−0.5	[−0.6, −0.3]	< 0.001***	0.0	[−0.5, 0.5]	0.900	0.2	[0.1, 0.4]	0.022*
	3LT versus Normal	−10.7	[−14.3, −7.2]	< 0.001***	1.2	[−0.2, 2.5]	0.145	−1.7	[−2.9, −0.5]	0.008**	−0.6	[−0.9, −0.3]	< 0.001***	1.0	[0.6, 1.5]	< 0.001***	0.2	[0.1, 0.4]	0.008**

*Note:* Displayed are the mean differences for the entirety of specified dynamic wrist motions between ligament deficient (injury) wrists compared to normal, and each surgical reconstruction technique (DTLT and 3LT) compared to normal. “Flexion,” “ulnar deviation,” and “ulnar flexion” correspond to the first quarter (0%−25%) of the wrist motion cycle, while “extension,” “radial deviation,” and “radial extension” correspond to the third quarter (50%–75%) of the cycle. All angles and translations are reported with respect to the radius. Mean differences along with a 95% confidence interval (CI) are given for each comparison. The asterisk indicates *p* < 0.05.

**Table 3:** Mean differences and *p*‐values for capitate Euler angle rotations (in degrees) and translations (in mm) across normal, ligament deficient, and surgically reconstructed wrists.

		Rotation [deg]									Translation [mm]								
		Flexion			Ulnar deviation			Pronation			Radial			Volar			Proximal		
		Mean diff.	95% CI	*p*‐value	Mean diff.	95% CI	*p*‐value	Mean diff.	95% CI	*p*‐value	Mean diff.	95% CI	*p*‐value	Mean diff.	95% CI	*p*‐value	Mean diff.	95% CI	*p*‐value
Flexion	Injury versus Normal	1.3	[0.5, 2.0]	0.009**	−0.4	[−0.7, −0.2]	0.003**	3.6	[2.5, 4.7]	< 0.001***	0.1	[−0.3, 0.4]	0.766	−2.2	[−3.2, −1.2]	< 0.001***	1.5	[1.1, 1.8]	< 0.001***
	DTLT versus Normal	0.0	[−0.5, 0.5]	0.353	0.4	[0.1, 0.6]	0.001**	−0.5	[−1.5, 0.5]	0.449	0.1	[−0.3, 0.5]	0.687	1.0	[0.4, 1.7]	0.004**	0.6	[0.4, 0.7]	< 0.001***
	3LT versus Normal	−0.3	[−0.8, 0.3]	0.394	−0.2	[−0.6, 0.2]	0.374	0.5	[−0.9, 1.8]	0.588	−1.1	[−1.3, −0.8]	< 0.001***	−1.4	[−2.2, −0.5]	0.008**	0.8	[0.5, 1.0]	< 0.001***
Extension	Injury versus Normal	1.3	[0.5, 2.2]	0.012*	−0.3	[−0.6, 0.1]	0.297	2.4	[−0.5, 5.3]	0.175	0.3	[−0.3, 0.8]	0.399	−1.6	[−3.5, 0.3]	0.168	1.4	[0.6, 2.1]	0.002**
	DTLT versus Normal	0.5	[0.1, 0.9]	0.045*	−0.3	[−0.7, 0.1]	0.279	−0.3	[−1.8, 1.2]	0.568	0.0	[−0.2, 0.3]	0.908	1.3	[0.7, 1.8]	< 0.001***	0.3	[0.2, 0.4]	< 0.001***
	3LT versus Normal	−0.4	[−1.0, 0.3]	0.291	−0.6	[−1.0, −0.3]	0.001**	−0.2	[−1.7, 1.3]	0.783	−0.8	[−0.9, −0.6]	< 0.001***	−1.1	[−1.8, −0.5]	0.005**	1.0	[0.7, 1.3]	< 0.001***
Ulnar deviation	Injury versus Normal	1.1	[0.3, 1.9]	0.022*	−0.5	[−0.9, −0.1]	0.043*	2.4	[0.7, 4.0]	0.017*	0.6	[0.2, 1.0]	0.022*	−1.5	[−2.6, −0.4]	0.020*	1.4	[0.9, 1.8]	< 0.001***
	DTLT versus Normal	0.6	[−0.1, 1.3]	0.172	0.0	[−0.3, 0.3]	0.547	0.5	[−0.8, 1.8]	0.305	0.2	[−0.1, 0.4]	0.360	1.7	[0.7, 2.8]	0.008**	0.2	[−0.1, 0.4]	0.214
	3LT versus Normal	−0.1	[−0.7, 0.4]	0.446	−0.7	[−1.1, −0.2]	0.009**	−0.2	[−1.9, 1.5]	0.817	−0.8	[−1.0, −0.6]	< 0.001***	−1.1	[−1.8, −0.4]	0.007**	1.0	[0.7, 1.3]	< 0.001***
Radial deviation	Injury versus Normal	1.6	[0.6, 2.5]	0.005**	−0.3	[−0.6, 0.1]	0.158	4.7	[3.6, 5.8]	< 0.001***	−0.5	[−0.8, −0.2]	0.001**	−1.5	[−2.5, −0.4]	0.023*	1.0	[0.7, 1.3]	< 0.001***
	DTLT versus Normal	0.7	[0.3, 1.0]	0.002**	−0.1	[−0.5, 0.2]	0.343	−0.1	[−0.8, 0.6]	0.581	0.0	[−0.3, 0.2]	0.786	0.9	[0.4, 1.4]	0.004**	0.5	[0.4, 0.5]	< 0.001***
	3LT versus Normal	0.4	[−0.3, 1.2]	0.356	−0.5	[−0.9, −0.2]	0.008**	1.2	[−0.3, 2.8]	0.169	−1.0	[−1.3, −0.8]	< 0.001***	−1.2	[−2.0, −0.4]	0.011*	0.9	[0.6, 1.2]	< 0.001***
DTM (ulnar flexion)	Injury versus Normal	0.6	[−0.1, 1.4]	0.149	−0.6	[−0.9, −0.2]	0.021*	1.6	[0.7, 2.6]	0.003**	0.2	[−0.1, 0.5]	0.210	−2.0	[−3.1, −0.9]	0.004**	1.0	[0.7, 1.3]	< 0.001***
	DTLT versus Normal	0.1	[−0.4, 0.7]	0.403	0.7	[0.5, 0.8]	< 0.001***	−0.5	[−1.7, 0.6]	0.410	0.0	[−0.4, 0.4]	0.640	1.8	[0.6, 2.9]	0.014*	0.4	[0.2, 0.7]	0.005**
	3LT versus Normal	−0.8	[−1.5, −0.1]	0.016*	−0.4	[−1.1, 0.2]	0.289	−0.5	[−1.9, 0.9]	0.402	−1.0	[−1.2, −0.7]	< 0.001***	−1.5	[−2.4, −0.6]	0.004**	0.7	[0.4, 0.9]	< 0.001***
DTM (radial extension)	Injury versus Normal	1.3	[0.1, 2.5]	0.096	−0.5	[−0.8, −0.2]	0.003**	2.6	[0.9, 4.2]	0.006**	−0.7	[−1.1, −0.4]	0.001**	−1.8	[−3.9, 0.3]	0.163	0.9	[0.6, 1.1]	< 0.001***
	DTLT versus Normal	0.6	[0.2, 1.1]	0.034*	−0.4	[−0.8, 0.0]	0.054	0.2	[−1.4, 1.7]	0.584	−0.1	[−0.4, 0.2]	0.602	1.1	[0.6, 1.6]	< 0.001***	0.3	[0.2, 0.5]	< 0.001***
	3LT versus Normal	−0.1	[−1.0, 0.8]	0.505	−0.6	[−0.7, −0.4]	< 0.001***	1.1	[−0.7, 2.9]	0.317	−0.9	[−1.1, −0.7]	< 0.001***	−1.0	[−1.7, −0.4]	0.010*	0.9	[0.6, 1.2]	< 0.001***

*Note:* Displayed are the mean differences for the entirety of specified dynamic wrist motions between ligament deficient (injury) wrists compared to normal, and each surgical reconstruction technique (DTLT and 3LT) compared to normal. “Flexion,” “ulnar deviation,” and “ulnar flexion” correspond to the first quarter (0%–25%) of the wrist motion cycle, while “extension,” “radial deviation,” and “radial extension” correspond to the third quarter (50%–75%) of the cycle. All angles and translations are reported with respect to the radius. Mean differences along with a 95% confidence interval (CI) are given for each comparison. The asterisk indicates *p* < 0.05.

### 3.2 DTLT

The DTLT technique restored normal scaphoid kinematics in the coronal plane (RUD) for all wrist motions, with no significant differences relative to that in the native wrist (*p* > 0.05), though this was not observed in other anatomical planes of motion. The scaphoid remained significantly more flexed during most wrist motions, with the greatest amount of flexion observed during wrist ulnar flexion compared to that in the healthy wrist (mean difference: 6.6°, *p* < 0.001). Moderate but statistically significant scaphoid pronation was observed, particularly during wrist radial (mean difference: 3.7°, *p* < 0.001) and ulnar deviation (mean difference: 3.9°, *p* = 0.05). DTLT corrected the radial and distal translation of the scaphoid in the ligament deficient wrist; however, this reconstruction resulted in significant volar translation of the scaphoid in all wrist motions (*p* < 0.001).

The significantly increased lunate extension, supination, and volar translation observed in the scapholunate ligament deficient wrist were reduced after the DTLT procedure, though increased lunate flexion was observed in most specimens with significant pronation during wrist ulnar deviation (*p* < 0.05). There was a persistent and significant ulnar translation of the lunate after the DTLT procedure, particularly during wrist extension (mean difference: 0.5mm, *p* < 0.001). The reconstruction restored the dynamic pattern of the radiolunate translation in the volar‐dorsal and proximal‐distal directions.

**Figure 6:**
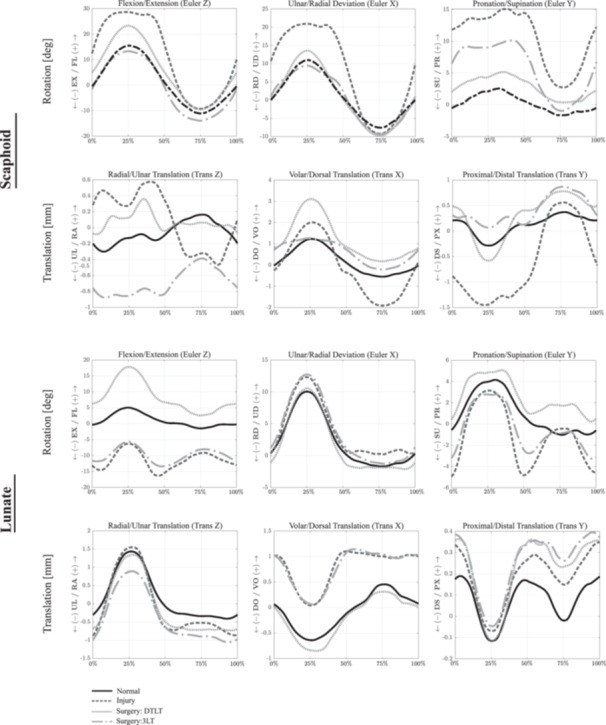
Mean scaphoid and lunate kinematics during wrist dart‐thrower's motion in the normal, ligament deficient and surgically reconstructed wrist following the DTLT and 3LT procedures. Given are the three Euler angle rotations (degrees), including flexion–extension (*Z*), ulnar‐radial deviation (*X*) and pronation‐supination (*Y*), as well as bone translations (mm). Data are given as a percentage of the sinusoidal wrist dart‐thrower's motion cycle. See Figure 4 Caption for more information.

No significantly increased pronation of the capitate was observed after the DTLT procedure (*p* > 0.05) (Figures 4, 5, 6). However, after reconstruction, there was a significant volar translation of the capitate in all wrist motions (*p* = 0.01). While lower than that in the scapholunate ligament deficient wrist (mean difference: 1.0 mm), the proximal translation of the capitate remained significantly larger than that in the healthy wrist following the DTLT procedure during wrist ulnar flexion (mean difference: 0.4 mm, *p* = 0.01).

### 3.3 3LT

The 3LT reconstruction restored normal scaphoid flexion and ulnar deviation for all wrist motions, with no significant differences in kinematics relative to that in the native wrist (*p* > 0.05). The procedure also restored the normal adaptive flexion and extension of the scaphoid observed in RUD. However, in the transverse plane, the scaphoid remained significantly pronated, especially during wrist ulnar flexion (mean difference: 7.6°, *p* < 0.001). The 3LT procedure also did not significantly change the scaphoid pronation and supination observed in the ligament deficient wrist, particularly during wrist extension (*p* > 0.05). Significant ulnar translation of the scaphoid relative to that in the healthy wrist was observed, particularly during wrist radial deviation (mean difference: 0.6mm, *p* = 0.01). There was a persistent significant volar translation of the scaphoid during wrist ulnar deviation (mean difference: 0.6mm, *p* < 0.001).

There remained significant extension (mean difference: 13.7°, *p* < 0.001) and supination (mean difference: 3.3°, *p* < 0.001) of the lunate in wrist radial deviation relative to that in the healthy intact wrist. There was significant volar and ulnar translation of the lunate especially during wrist ulnar deviation (mean difference:0.8mm, *p* < 0.001). After the 3LT procedure, the pathological flexion and pronation of the capitate in the scapholunate ligament deficient wrist was restored, with no significant difference in kinematics relative to that in the healthy wrist (*p* > 0.05). However, there was a significantly persistent ulnar translation of the capitate relative to that in the normal wrist (mean difference: 1.0mm, *p* < 0.001).

## 4 Discussion

This study examined 3D carpal bone kinematics in the intact, d‐SLIL deficient, and surgically reconstructed wrist by employing bi‐plane fluoroscopy and a dynamic wrist simulator capable of simulating active wrist motions using physiologically relevant muscle forces, including muscle co‐contraction, and soft‐tissue deformation. Reconstruction of the scapholunate deficient wrist with the DTLT and 3LT procedures restored normal kinematics of the scaphoid, lunate, and capitate to an extent. The DTLT procedure corrected the increased scaphoid ulnar deviation and pronation but did not improve the scaphoid flexion and resulted in volar translation of the scaphoid. The 3LT procedure restored scaphoid flexion and ulnar deviation but did not correct pronation and also failed to correct the increased lunate extension and supination, and the volar and ulnar translation observed in the ligament deficient wrist.

We showed that while the DTLT procedure mitigated abnormal scaphoid motion in the coronal plane (RUD) in the scapholunate ligament deficient wrist, it did not correct the abnormal scaphoid flexion seen after the complete division of the primary and secondary stabilizers of the scapholunate joint. This may be due to the position of the tendon grafts used in this technique, which in contrast to the tendon graft in the 3LT procedure, do not restrain the distal pole of the scaphoid and hence do not limit scaphoid flexion. The DTLT reconstruction caused further flexion of the scaphoid as the wrist was moved into flexion or ulnar flexion which was likely due to the nature of the ECR‐B graft remaining attached to the third metacarpal. An approach to correct this scaphoid flexion may be to perform a dorsal capsulodesis by attaching the trapezium and trapezoid insertion of the DIC onto the dorsum of the distal scaphoid [10] or by releasing the graft from the third metacarpal. In the present study, reattaching the distal limb of the DIC was not possible due to its release, which was necessary for the release of this secondary stabilizer.

In the present study, the 3LT procedure stabilized the scaphoid more effectively in the sagittal plane compared to the DTLT procedure, correcting the pathological increase in flexion of the scaphoid and the dynamic radioscaphoid rotation observed in the d‐SLIL deficient wrist. This included restoration of abnormal radioscaphoid flexion and extension in wrist RUD. These findings may be attributed to the reconstruction of the STT ligament, as the FCR tendon graft is passed through the scaphoid tubercle. In the transverse plane, however, the 3LT did not correct scaphoid pronation. It was also unable to correct dynamic scaphoid supination during wrist extension and radial extension, suggesting a dorsally pivoted rotation of the scaphoid and indicative of a potential volar opening of the scapholunate interval. This phenomenon may be attributed to passive stretching of the FCR graft during wrist extension since the radially situated graft passed obliquely to the dorsum of the scaphoid and was tensioned around the DRC ligament. The ulnar and dorsal directed moment generated as a result of the 3LT reconstruction is likely to be in the plane of scaphoid pronation, which may exacerbate carpal malalignment in the ligament deficient wrist. This may be addressed by reconstruction of the volar component of the scapholunate ligament (v‐SLIL) in conjunction with the 3LT, similar to that described by Corella et al. [28].

The 3LT procedure did not effectively mitigate dorsal intercalated segment instability (DISI) and restore normal kinematics of the lunate. This failure to reduce lunate extension using the 3LT or other variants of the Brunelli technique has been observed in clinical studies. In a retrospective analysis of static scapholunate instability in six patients, Moran et al. [29] reported that the RL angle improved from 16° pre‐operatively to 14° post‐operatively. Chabas et al. [30] also observed that d‐SLIL reconstruction only led to a minor immediate correction of the RL angle and recurrence of the DISI deformity was observed in 26% of patients. Pauchard et al. [31] found that the radiolunate angle worsened following the 3LT in patients diagnosed with dynamic carpal instability and reported no appreciable improvement in those with static DISI deformity. The increased lunate supination observed in this study during wrist radial deviation, and ulnar translation of the carpus after the reconstruction, is likely related to the tethering of the tendon graft to the DRC. Excessive tightening of the tendon graft as it passes around the DRC ought to be avoided, for example, by passing the graft through the radial half of the DRC rather than through its ulnar border. Ideally, placement of the tunnel, through which the FCR graft passes through the scaphoid, should be directed to correct excessive scaphoid pronation.

The carpal bone kinematic study of Zhang et al. [25] demonstrated significant static and kinematic changes to the scaphoid and lunate after division of the dorsal component of the SLIL and dorsal extrinsic ligaments (DIC, DRC, and volar STT ligaments). As the division of the volar and membranous components of the SLIL did not significantly alter carpal kinematics, reconstruction of the v‐SLIL may not be justified. As the integrity of the attachments of the extrinsic ligaments is functionally relevant, ligament‐sparing approaches should be considered, and if a deficiency of these ligaments is identified, they ought to be repaired or form part of the reconstructive ladder. Correcting the static alignment of the scaphoid and lunate is an important first step in the surgical management of the scapholunate deficient wrist, as a persisting DISI alignment would impose increased loading on the dorsal articular surface of the luno‐capitate joint. Ultimately, persistent abnormal scaphoid kinematics after reconstruction may cause the onset and progression of wear in the concave scaphoid fossa.

The finding that reconstruction of the ligament deficient wrist does not completely restore normal wrist kinematics has been alluded to in a number of past studies. By employing a wrist simulation device and electromagnetic motion sensors, Short, Werner, and Sutton [32] measured scaphoid and lunate kinematics in eight cadaver wrists after incremental ligament division and following a subsequent reconstruction using the DIC ligament. They concluded that an isolated repair of this ligament is inadequate for stabilizing the scaphoid and lunate after observing the reoccurrence of the instability along with gapping of the scapholunate interval within a few cycles of wrist motion. Lee et al. [33] compared the radiographic outcomes of three different techniques, including the scapholunate axis method (SLAM) reconstruction, the Blatt capsulodesis, and the modified Brunelli tenodesis, in a total of 12 cadaveric specimens. The authors manually cycled each specimen between flexion and extension and found that the SLAM reconstruction trended toward greater restoration of both the native scapholunate angle and interval. More recently, Loisel et al. [34] studied the efficacy of a direct repair of the scapholunate ligament by means of flexible anchors in three contralateral pairs of specimens. By utilizing a biplane X‐ray and a passive wrist simulator, the authors demonstrated a 7.4° reduction in pathological scaphoid flexion after an isolated dorsal repair and a further 2.9° reduction after volar repair, underscoring the potential efficacy of combined surgical approaches.

The normal and injured SL kinematics reported in the present study are largely consistent with existing research, establishing a foundation for the evaluation of surgical reconstructions. Specifically, the measured radioscaphoid and radiolunate kinematics in normal specimens are in close agreement with those reported by Kobayashi et al. [35], Moojen et al. [36], Goto et al. [37], and Stoesser et al. [23], who employed a similar experimental setup (see Zhang et al. [25] for details). For the ligament deficient wrist, our general trends in altered scapholunate kinematics agree with those reported by Short et al. [38]; however, the magnitude of the changes observed following injury was greater in the present study compared to those reported by Padmore et al. [39]. This discrepancy could be attributed to the physiologically relevant forces in the present study, which included muscle co‐contraction, and were higher in magnitude.

This study has a number of limitations. First, to ensure the resultant joint motions were repeatable and consistently stable across all specimens following simulated scapholunate disruption and subsequent reconstructions, wrist motion simulations were kept within a narrow range at a lower frequency of 0.05Hz. The carpal kinematics reported may be different to those occurring at higher speeds. Future studies ought to consider higher‐speed carpal bone motion analysis through a larger range of motion, and this could be supported using optical motion analysis methods. 4D‐CT might ultimately be used for in vivo motion analysis in ligament deficient and surgically reconstructed wrists, though with lower sample rates and ionizing radiation which requires ethical consideration. Second, the cadaveric specimens were of advanced age, and the injury model reported is thus representative of an older demographic. The mechanical properties of the ligament may be different from those of a younger adult cohort, which could influence carpal kinematics. Third, the present study constrained the range of motion to a maximum of 30° of flexion and extension following the protocol of others [40, 41, 42], which also served to mitigate retro‐reflective marker occlusion during testing. As a consequence, the carpal kinematics may have been more midcarpal dominated with less radiocarpal motion. While this constrained motion arc is relevant to limited motion range in patients recovering from reconstructive surgery, future studies ought to explore carpal kinematics through a broader range of global wrist motion. Fourth, the DRC was only partially divided to accommodate the 3LT procedure, and the limited specimen availability precluded testing other permutations of the surgical reconstructions. Fifth, the 3LT procedure was performed before DTLT; however, the carpal kinematics results may be sequence‐dependent. Furthermore, some small magnitude but significant differences in kinematics between conditions may not be clinically relevant and ought to be interpreted with caution. Analysis of peaks in motion profiles was not performed, and in the future, could supplement the dataset presented and provide an alternative interpretation of the study findings. Finally, the findings may differ if carpal kinematics were reported as a function of third metacarpal bone motion due to the presence of hysteresis, since carpal kinematics depends not only on wrist motion in a given plane but the direction of that wrist motion. For example, carpal kinematics when moving from wrist flexion to extension differs from that moving from extension to flexion (see Supporting Information: S1).

In conclusion, static realignment of the scaphoid and lunate in the d‐SLIL deficient wrist can be achieved after reconstruction using the DTLT and 3LT techniques, but the resulting dynamic carpal motion is only partially improved. Substantial pathological kinematics of the scaphoid, lunate and capitate kinematics remain after reconstruction using both techniques. These findings, which provide evidence of the functional performance of reconstructive techniques, suggest that d‐SLIL repair may not completely address all structures injured nor mitigate long‐term degenerative joint conditions at the wrist. The outcomes of this study may be useful in the planning of wrist reconstructive surgery.

## Author Contributions

**Xin Zhang:** methodology, validation, formal analysis, writing–original draft, investigation. **Stephen K. Tham:** conceptualization, writing–review and editing, funding acquisition. **Bruno Crepaldi:** data curation, investigation. **Eugene T. Ek:** conceptualization, writing–review and editing, funding acquisition. **David McCombe:** conceptualization, writing–review and editing, funding acquisition. **David Charles Ackland:** conceptualization, resources, supervision, project administration, funding acquisition. All authors have read and approved the final submitted manuscript.

## Conflicts of Interest

The authors declare no conflicts of interest.

## Supporting information

Supporting information.

## References

[1] A. Kitay and S. W. Wolfe , “Scapholunate Instability: Current Concepts in Diagnosis and Management,” Journal of Hand Surgery 37, no. 10 (2012): 2175–2196. 23021178 10.1016/j.jhsa.2012.07.035

[2] W. A. Jones , “Beware the Sprained Wrist. The Incidence and Diagnosis of Scapholunate Instability,” Journal of Bone and Joint Surgery British Volume 7–B, no. 2 (1988): 293–297. 10.1302/0301-620X.70B2.3346308 3346308

[3] I. P. Pappou , J. Basel , and D. N. Deal , “Scapholunate Ligament Injuries: A Review of Current Concepts,” Hand 8, no. 2 (2013): 146–156. 24426911 10.1007/s11552-013-9499-4 PMC3653000

[4] H. K. Watson and F. L. Ballet , “The SLAC Wrist: Scapholunate Advanced Collapse Pattern of Degenerative Arthritis,” Journal of Hand Surgery 9, no. 3 (1984): 358–365. 6725894 10.1016/s0363-5023(84)80223-3

[5] W. B. Kleinman , J. B. Steichen , and J. W. Strickland , “Management of Chronic Rotary Subluxation of the Scaphoid by Scapho‐Trapezio‐Trapezoid Arthrodesis,” Journal of Hand Surgery 7, no. 2 (1982): 125–136. 7069167 10.1016/s0363-5023(82)80076-2

[6] H. K. Watson and J. Ryu , “Evolution of Arthritis of the Wrist,” Clinical Orthopaedics and Related Research 202, no. 202 (1986): 57–67. 3955970

[7] M. Garcia‐Elias , W. P. Cooney , K. N. An , R. L. Linscheid , and E. Y. S. Chao , “Wrist Kinematics After Limited Intercarpal Arthrodesis,” Journal of Hand Surgery 14, no. 5 (1989): 791–799. 2794393 10.1016/s0363-5023(89)80077-2

[8] A. M. Holleran , R. J. Quigley , G. H. Rafijah , and T. Q. Lee , “Radioscapholunate Arthrodesis With Excision of the Distal Scaphoid: Comparison of Contact Characteristics to the Intact Wrist,” Journal of Hand Surgery 38, no. 4 (2013): 706–711. 23474154 10.1016/j.jhsa.2013.01.035

[9] C. F. Larsen , R. A. Jacoby , and S. J. McCabe , “Nonunion Rates of Limited Carpal Arthrodesis: A Meta‐Analysis of the Literature,” Journal of Hand Surgery 22, no. 1 (1997): 66–73. 9018614 10.1016/S0363-5023(05)80181-9

[10] R. M. Szabo , R. R. Slater , C. F. Palumbo , and T. Gerlach , “Dorsal Intercarpal Ligament Capsulodesis for Chronic, Static Scapholunate Dissociation: Clinical Results,” Journal of Hand Surgery 27, no. 6 (2002): 978–984. 12457347 10.1053/jhsu.2002.36523

[11] C. J. Lavernia , M. S. Cohen , and J. Taleisnik , “Treatment of Scapholunate Dissociation by Ligamentous Repair and Capsulodesis,” Journal of Hand Surgery 17, no. 2 (1992): 354–359. 1564287 10.1016/0363-5023(92)90419-p

[12] S. C. Deshmukh , P. Givissis , D. Belloso , J. K. Stanley , and I. A. Trail , “Blatt's Capsulodesis for Chronic Scapholunate Dissociation,” Journal of Hand Surgery 24, no. 2 (1999): 215–220. 10372779 10.1054/jhsb.1998.0183

[13] S. L. Moran , W. P. Cooney , R. A. Berger , and J. Strickland , “Capsulodesis for the Treatment of Chronic Scapholunate Instability,” Journal of Hand Surgery 30, no. 1 (2005): 16–23. 15680551 10.1016/j.jhsa.2004.07.021

[14] S. J. Svoboda , W. A. Eglseder , and S. M. Belkoff , “Autografts From the Foot for Reconstruction of the Scapholunate Interosseous Ligament,” Journal of Hand Surgery 20, no. 6 (1995): 980–985. 8583071 10.1016/S0363-5023(05)80146-7

[15] A. P. C. Weiss , “Scapholunate Ligament Reconstruction Using a Bone‐Retinaculum‐Bone Autograft,” Journal of Hand Surgery 23, no. 2 (1998): 205–215. 9556257 10.1016/S0363-5023(98)80115-9

[16] E. J. Harvey , R. A. Berger , A. L. Osterman , D. L. Fernandez , and A. P. Weiss , “Bone‐Tissue‐Bone Repairs for Scapholunate Dissociation,” Journal of Hand Surgery 32, no. 2 (2007): 256–264. 10.1016/j.jhsa.2006.11.011 17275604

[17] M. P. Rosenwas , K. C. Miyasaka , and R. J. Strauch , “The RASL Procedure: Reduction and Association of the Scaphoid and Lunate Using the Herbert Screw,” Techniques in Hand & Upper Extremity Surgery 1, no. 4 (1997): 263–272. 16609495

[18] S. M. Pisano , C. A. Peimer , D. R. Wheeler , and F. Sherwin , “Scaphocapitate Intercarpal Arthrodesis,” Journal of Hand Surgery 16, no. 2 (1991): 328–333. 2022848 10.1016/s0363-5023(10)80121-2

[19] V. K. Gajendran , B. Peterson , R. R. Slater Jr. , and R. M. Szabo , “Long‐Term Outcomes of Dorsal Intercarpal Ligament Capsulodesis for Chronic Scapholunate Dissociation,” Journal of Hand Surgery 32, no. 9 (2007): 1323–1333. 17996765 10.1016/j.jhsa.2007.07.016

[20] G. A. Brunelli and G. R. Brunelli , “A New Technique to Correct Carpal Instability With Scaphoid Rotary Subluxation: A Preliminary Report,” Journal of Hand Surgery 20, no. 3, Part 2 (1995): S82–S85. 7642955 10.1016/s0363-5023(95)80175-8

[21] M. Garcia‐Elias , A. L. Lluch , and J. K. Stanley , “Three‐Ligament Tenodesis for the Treatment of Scapholunate Dissociation: Indications and Surgical Technique,” Journal of Hand Surgery 31, no. 1 (2006): 125–134. 16443117 10.1016/j.jhsa.2005.10.011

[22] P. C. Zarkadas , P. T. Gropper , N. J. White , and B. H. Perey , “A Survey of the Surgical Management of Acute and Chronic Scapholunate Instability,” Journal of Hand Surgery 29, no. 5 (2004): 848–857. 15465234 10.1016/j.jhsa.2004.05.008

[23] H. Stoesser , C. Padmore , M. Nishiwaki , B. Gammon , G. Langohr , and J. Johnson , “Biomechanical Evaluation of Carpal Kinematics During Simulated Wrist Motion,” Journal of Wrist Surgery 06, no. 2 (2017): 113–119. 10.1055/s-0036-1588025 PMC5397313 28428912

[24] R. A. Berger , A. T. Bishop , and P. C. Bettinger , “New Dorsal Capsulotomy for the Surgical Exposure of the Wrist,” Annals of Plastic Surgery 35, no. 1 (1995): 54–59. 7574287 10.1097/00000637-199507000-00011

[25] X. Zhang , S. Tham , E. T. Ek , D. McCombe , and D. C. Ackland , “Scaphoid, Lunate and Capitate Kinematics in the Normal and Ligament Deficient Wrist: A Bi‐Plane X‐Ray Fluoroscopy Study,” Journal of Biomechanics 158 (2023): 111685. 37573806 10.1016/j.jbiomech.2023.111685

[26] G. Wu , F. C. T. van der Helm , H. E. J. DirkJan Veeger , et al., “ISB Recommendation on Definitions of Joint Coordinate Systems of Various Joints for the Reporting of Human Joint Motion—Part II: Shoulder, Elbow, Wrist and Hand,” Journal of Biomechanics 38, no. 5 (2005): 981–992. 15844264 10.1016/j.jbiomech.2004.05.042

[27] T. Górecki and Ł. Smaga , “FdANOVA: An R Software Package for Analysis of Variance for Univariate and Multivariate Functional Data,” Computational Statistics 34, no. 2 (2019): 571–597.

[28] F. Corella , M. Del Cerro , M. Ocampos , and R. Larrainzar‐Garijo , “Arthroscopic Ligamentoplasty of the Dorsal and Volar Portions of the Scapholunate Ligament,” Journal of Hand Surgery 38, no. 12 (2013): 2466–2477. 24275054 10.1016/j.jhsa.2013.09.021

[29] S. L. Moran , K. S. Ford , C. A. Wulf , and W. P. Cooney , “Outcomes of Dorsal Capsulodesis and Tenodesis for Treatment of Scapholunate Instability,” Journal of Hand Surgery 31, no. 9 (2006): 1438–1446. 17095371 10.1016/j.jhsa.2006.08.002

[30] J. F. Chabas , A. Gay , D. Valenti , D. Guinard , and R. Legre , “Results of the Modified Brunelli Tenodesis for Treatment of Scapholunate Instability: A Retrospective Study of 19 Patients,” Journal of Hand Surgery 33, no. 9 (2008): 1469–1477. 18984325 10.1016/j.jhsa.2008.05.031

[31] N. Pauchard , A. Dederichs , J. Segret , S. Barbary , F. Dap , and G. Dautel , “The Role of Three‐Ligament Tenodesis in the Treatment of Chronic Scapholunate Instability,” Journal of Hand Surgery (European Volume) 38, no. 7 (2013): 758–766. 23400768 10.1177/1753193413475753

[32] W. H. Short , F. W. Werner , and L. G. Sutton , “Dynamic Biomechanical Evaluation of the Dorsal Intercarpal Ligament Repair for Scapholunate Instability,” Journal of Hand Surgery 34, no. 4 (2009): 652–659. 19345867 10.1016/j.jhsa.2008.12.009

[33] S. K. Lee , D. A. Zlotolow , A. Sapienza , R. Karia , and J. Yao , “Biomechanical Comparison of 3 Methods of Scapholunate Ligament Reconstruction,” Journal of Hand Surgery 39, no. 4 (2014): 643–650. 24559758 10.1016/j.jhsa.2013.12.033

[34] F. Loisel , S. Durand , S. Persohn , et al., “Scapholunate Kinematics After Flexible Anchor Repair,” Medical Engineering & Physics 75 (2020): 59–64. 31734015 10.1016/j.medengphy.2019.11.001

[35] M. Kobayashi , R. A. Berger , L. Nagy , et al., “Normal Kinematics of Carpal Bones: A Three‐Dimensional Analysis of Carpal Bone Motion Relative to the Radius,” Journal of Biomechanics 30, no. 8 (1997): 787–793. 9239563 10.1016/s0021-9290(97)00026-2

[36] T. M. Moojen , J. G. Snel , M. J. P. F. Ritt , J. M. G. Kauer , H. W. Venema , and K. E. Bos , “Three‐Dimensional Carpal Kinematics In Vivo,” Clinical Biomechanics 17, no. 7 (2002): 506–514. 12206941 10.1016/s0268-0033(02)00038-4

[37] A. Goto , H. Moritomo , T. Murase , et al., “In Vivo Three‐Dimensional Wrist Motion Analysis Using Magnetic Resonance Imaging and Volume‐Based Registration,” Journal of Orthopaedic Research 23, no. 4 (2005): 750–756. 16022986 10.1016/j.orthres.2004.10.001

[38] W. H. Short , F. W. Werner , J. K. Green , and S. Masaoka , “Biomechanical Evaluation of the Ligamentous Stabilizers of the Scaphoid and Lunate: Part II,” Journal of Hand Surgery 30, no. 1 (2005): 24–34. 15680552 10.1016/j.jhsa.2004.09.015

[39] C. Padmore , H. Stoesser , G. D. Langohr , J. Johnson , and N. Suh , “Carpal Kinematics Following Sequential Scapholunate Ligament Sectioning,” Journal of Wrist Surgery 08, no. 2 (2019): 124–131. 10.1055/s-0038-1676865 PMC6443536 30941252

[40] F. W. Werner , W. H. Short , and J. K. Green , “Changes in Patterns of Scaphoid and Lunate Motion During Functional Arcs of Wrist Motion Induced by Ligament Division,” Journal of Hand Surgery 30, no. 6 (2005): 1156–1160. 16344171 10.1016/j.jhsa.2005.08.005 PMC1986800

[41] S. Erhart , M. Lutz , R. Arora , and W. Schmoelz , “Measurement of Intraarticular Wrist Joint Biomechanics With a Force Controlled System,” Medical Engineering & Physics 34, no. 7 (2012): 900–905. 22035674 10.1016/j.medengphy.2011.10.003

[42] J. Eschweiler , J. P. Stromps , B. Rath , N. Pallua , and K. Radermacher , “Analysis of Wrist Bone Motion Before and After SL‐Ligament Resection,” Biomedical Engineering/Biomedizinische Technik 61, no. 3 (2016): 345–357. 26402881 10.1515/bmt-2014-0167

