# Supplementary material for: Carpal Kinematics in the Normal, Scapholunate Ligament Deficient, and Surgically Reconstructed Wrist
Source: J Orthop Res. 2025 Feb 2;43(4):756–69. doi: 10.1002/jor.26049 (PMC11898162; doi:10.1002/jor.26049)
Supplement: Supplementary file 1 — Supporting information. [file JOR-43-756-s001.docx]

**SUPPLEMENTARY MATERIAL**


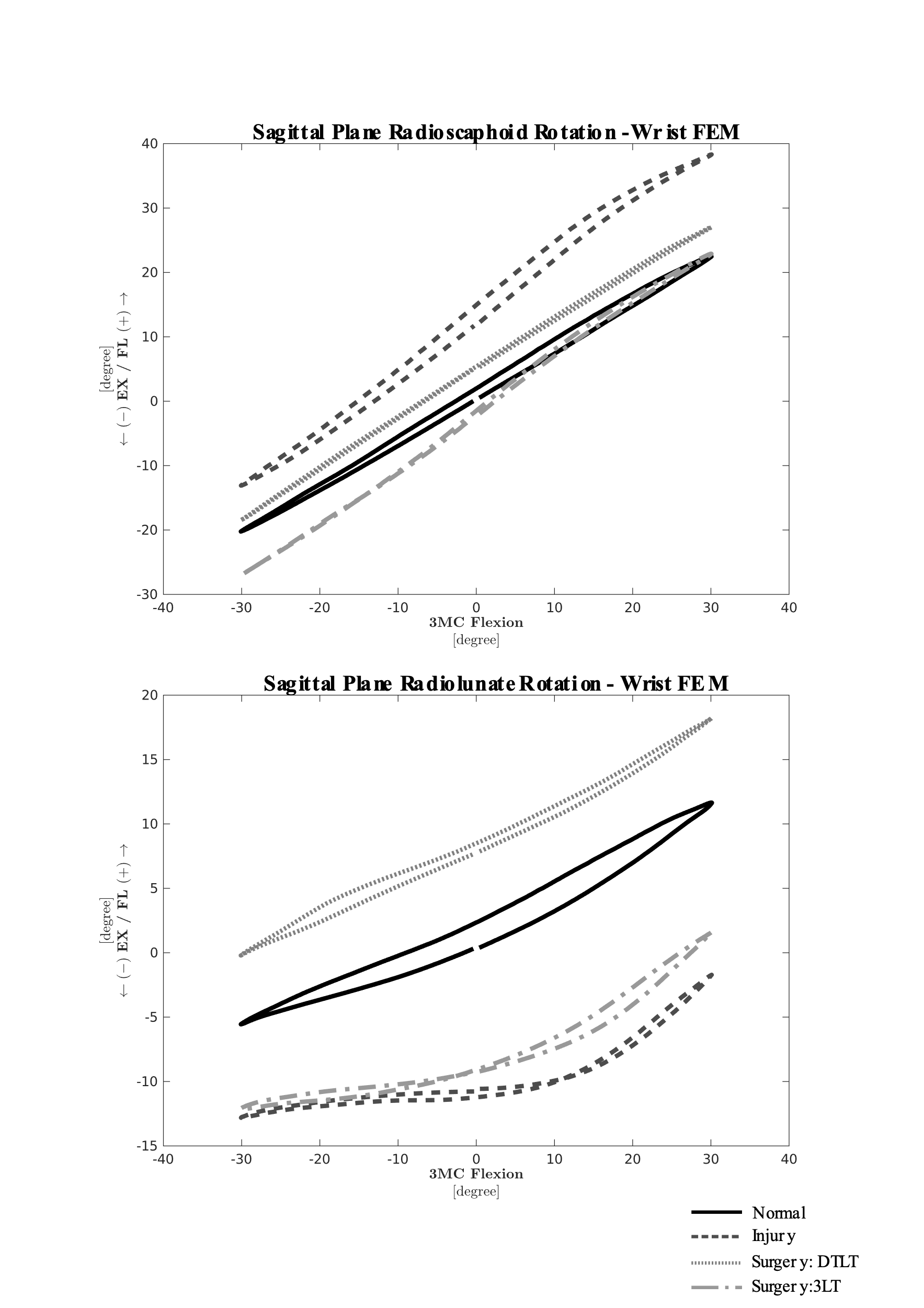
Figure S1: Illustration of hysteresis from radioscaphoid (top chart) and radiolunate (bottom chart) rotation during wrist flexion-extension motion (FEM). Hysteresis is observed from the different carpal kinematics motion path when the wrist moves from flexion to extension as compared to moving from extension to flexion.


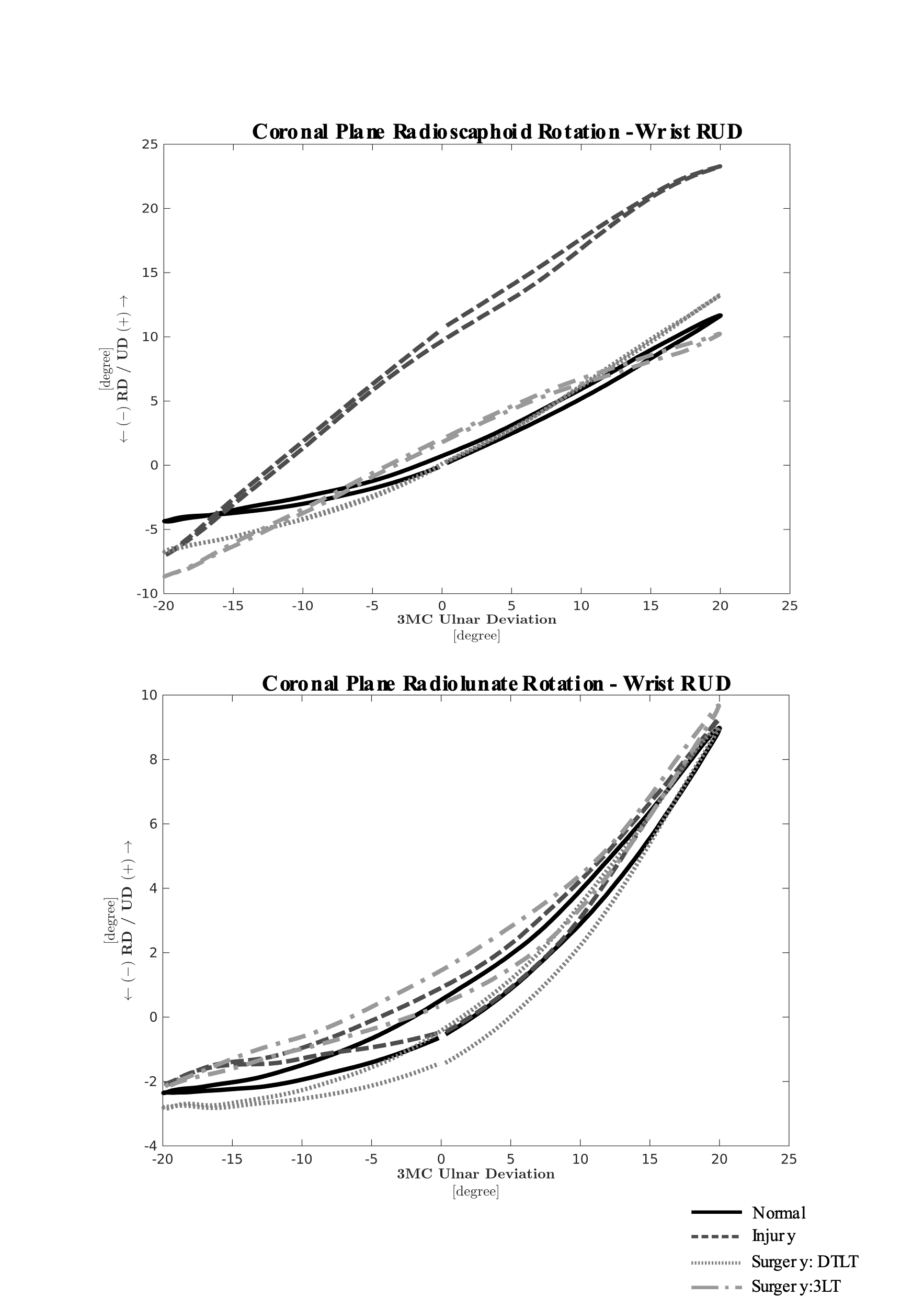
Figure S2: Illustration of hysteresis from radioscaphoid (top chart) and radiolunate (bottom chart) rotation during wrist radial-ulnar deviation motion (RUD). Hysteresis is observed from the different carpal kinematics motion path when the wrist moves from flexion to extension as compared to moving from extension to flexion.
